# Profiling of Antifungal Activities and In Silico Studies of Natural Polyphenols from Some Plants

**DOI:** 10.3390/molecules26237164

**Published:** 2021-11-26

**Authors:** Beenish Khanzada, Nosheen Akhtar, Mohammad K. Okla, Saud A. Alamri, Abdulrahman Al-Hashimi, Muhammad Waleed Baig, Samina Rubnawaz, Hamada AbdElgawad, Abdurahman H. Hirad, Ihsan-Ul Haq, Bushra Mirza

**Affiliations:** 1 Institute of Biochemistry, University of Sindh, Jamshoro 76080, Pakistan; beenish@usindh.edu.pk; 2 Department of Biochemistry, Quaid-i-Azam University, Islamabad 45320, Pakistan; samina.r.nawaz@gmail.com; 3 Department of Biological Sciences, National University of Medical Sciences, Rawalpindi 46000, Pakistan; 4 Botany and Microbiology Department, College of Science, King Saud University, Riyadh 11451, Saudi Arabia; malokia@ksu.edu.sa (M.K.O.); saualamri@ksu.edu.sa (S.A.A.); aalhashimi@ksu.edu.sa (A.A.-H.); hiirad1@gmail.com (A.H.H.); 5 Department of Pharmacy, Quaid-i-Azam University, Islamabad 45320, Pakistan; mwbg7@yahoo.com (M.W.B.); ihaq@qau.edu.pk (I.-U.H.); 6 Integrated Molecular Plant Physiology Research, Department of Biology, University of Antwerp, 2020 Antwerpen, Belgium; hamada.abdelgawad@uantwerpen.be

**Keywords:** antifungal, edible plants, molecular docking, antioxidant activities, polyphenols

## Abstract

A worldwide increase in the incidence of fungal infections, emergence of new fungal strains, and antifungal resistance to commercially available antibiotics indicate the need to investigate new treatment options for fungal diseases. Therefore, the interest in exploring the antifungal activity of medicinal plants has now been increased to discover phyto-therapeutics in replacement to conventional antifungal drugs. The study was conducted to explore and identify the mechanism of action of antifungal agents of edible plants, including *Cinnamomum zeylanicum, Cinnamomum tamala, Amomum subulatum, Trigonella foenumgraecum, Mentha piperita, Coriandrum sativum, Lactuca sativa,* and *Brassica oleraceae var. italica*. The antifungal potential was assessed via the disc diffusion method and, subsequently, the extracts were assessed for phytochemicals and total antioxidant activity. Potent polyphenols were detected using high-performance liquid chromatography (HPLC) and antifungal mechanism of action was evaluated in silico. *Cinnamomum zeylanicum* exhibited antifungal activity against all the tested strains while all plant extracts showed antifungal activity against *Fusarium solani*. Rutin, kaempferol, and quercetin were identified as common polyphenols. In silico studies showed that rutin displayed the greatest affinity with binding pocket of fungal 14-alpha demethylase and nucleoside diphosphokinase with the binding affinity (K_d_, −9.4 and −8.9, respectively), as compared to terbinafine. Results indicated that *Cinnamomum zeylanicum* and *Cinnamomum tamala* exert their antifungal effect possibly due to kaempferol and rutin, respectively, or possibly by inhibition of nucleoside diphosphokinase (NDK) and 14-alpha demethylase (CYP51), while *Amomum subulatum* and *Trigonella foenum graecum* might exhibit antifungal potential due to quercetin. Overall, the study demonstrates that plant-derived products have a high potential to control fungal infections.

## 1. Introduction

Fungal infections are continuously on the rise and one of the major causes of morbidity and mortality, particularly in immune-compromised patients [1,2]. Among the most predominant fungal infections are those caused by *Candida*, *Aspergillus*, *Fusarium species* (spp.), and *Mucor* spp. [2]. A fungus, such as *Fusarium* spp., produces mycotoxins that can cause mycotoxicosis in humans upon ingestion of food, having a colony of this fungus. *Fusarium solani* induces skin lesions and onychomycosis [3]. *Aspergillus* spp. mediated aspergillosis is increasing among patients undergoing chemotherapy and in patients with low immunity [4]. Moreover, *Mucor* spp. mediated mucormycosis is also widely reported [5]. For the treatment of fungal infections, four types of antibiotics are often offered, i.e., azole (Fluconazole), polyenes (Amphotericin B), echinocandins (caspofungin), flucytosine (5 fluorocytosine) [6], all offering a different mode of action. Terbinafine inhibits squalene epoxidase (fungal cell wall enzyme involved in ergosterol biosynthesis pathway) while azoles mostly inhibit 14-alpha demethylase [7,8]. Despite increased awareness and improved treatment strategies, drug resistance among fungal pathogens is enduring to develop, leading serious threat to public health and healthcare systems, worldwide. Dependence on antifungal antibiotics and their recurrent doses might lead to the development of resistance, as recently reported by Doung et al. in the case of itraconazole resistant isolates of *Aspergillus flavus* [9]. Moreover, many antifungal agents have been reported for complications in host tissue [8]. For instance, Aspergillosis is mostly treated with azoles but hepatotoxicity and visual disturbance are seen as side effects [10]. Hence, there is an urgent need to find novel agents to treat fungal infections with greater antifungal activity and fewer side effects.

To protect themselves against pathogens, plants produce and exude a myriad of secondary metabolites, which play important roles as defense mechanisms against various infections. The approach of traditional medicinal plant therapy is preferable due to fewer side effects and better efficacy [11]. The use of phytochemicals, either alone or in combination with conventional drugs, could be a better solution for fungal infections, due to reduced toxicity and minimum environmental impact [12]. In this connection, alcoholic extracts of medicinal plants could offer a better extraction, as some of the alcoholic extracts have been found to act as a more functional antifungal drug than conventional antibiotics [13]. Antifungal activity of aqueous extracts has also been observed and reported extensively [13]. Among phytochemicals, polyphenols have been reported to have antifungal effects against various fungal pathogens either by plasma membrane disruption, inhibition of cell wall and DNA/RNA/protein synthesis, and mitochondrial dysfunction [6]. For instance, quercetin, myricetin, and naringenin exhibit antifungal effects against *Candida albicans* and *Saccharomyces cerevisiae* [14]. Elagic acid and caffeic acid are recently reported to inhibit *Candida auris* by modifying fungal cell wall [15]. Another polyphenol, curcumin has shown a synergistic effect with itraconazole, co-trimoxazole, and amphotericin B by enhancing the reactive oxygen species production [16]. Nowadays, in silico tools are being used as an efficient method to predict the anti-microbial efficacy of phytobiotics [17]. In this regard, target identification for specific polyphenol should be focused for developing plant based drugs. For instance, superoxide dismutase, catalase, and isocitrate lyase have been proposed as molecular targets for curcumin via in silico studies [16]. Likewise, other plant polyphenols should be assessed for targeting specific fungal enzymes.

Hence, assessment of the medicinal plants for antifungal potential should be prioritized, and the mechanism of action should be focused to discover novel phyto-therapeutic agents. In this contribution, we selected eight plants, including *Cinnamomum zeylanicum (C. zeylanicum), Cinnamomum tamala (C. tamala), Amomum subulatum (A. subulatum), Trigonella foenumgraecum (T. foenumgraecum), Mentha piperita (M. piperita), Coriandrum sativum (C. sativum), Lactuca sativa (L. sativa),* and *Brassica oleraceae var. italica (B. oleraceae)*) and evaluated their antifungal potential. These common plants were selected due to their minimal/nontoxicity, significant antioxidant and antimicrobial properties, and their frequent use in routine diet. Active phytochemicals of plants were quantified using high-performance liquid chromatography (HPLC). Furthermore, in silico studies were performed to comprehend the underlying antifungal mechanism of detected polyphenols.

## 2. Results

### 2.1. Percent Extract Recovery

Percent extract yield for selected herbs and spices was calculated, as shown in Table 1. Among spices, *C. zeylanicum* has the highest percent yield, while *M. piperita* presented a high percent recovery among herbs.

### 2.2. Antifungal Activity

The antifungal potential of extracts was assessed against four pathogenic fungal strains. Results showed that all studied plant extracts inhibited the growth of *Fusarium solani*. *C. zeylanicum*) inhibited the growth of all fungal strains with a maximum zone of inhibition (13.6 mm) against *Fusarium solani*. *A. subulatum* exhibited antifungal activity against *Fusarium solani*, *Aspergillus niger,* and *Aspergillus flavus*. Overall, extracts of *T. foenumgraecum*, *C. zeylanicum, C. tamala*, and *A. subulatum* showed greater antifungal activity against *Fusarium solani* than herbal extracts, i.e., *C. sativum, L. sativa, B. oleraceae,* and *M. piperita* (7.6 to 10 mm), as shown in Table 2. No other extract inhibited the growth of *Aspergillus flavus* except *C. zeylanicum* and *A. subulatum*. *M. piperita* and *T. foenumgraecum* exhibited antifungal activity against *Fusarium solani* and *Aspergillus niger* while *B. oleraceae* possessed antifungal potential against *Fusarium solani* and *Mucor spp. C. sativum* and *L. sativa* exhibited antifungal potential only against *Fusarium solani* (Table 2). Terbinafine, the standard antifungal drug, showed significant antifungal potential against all the fungal strains.

### 2.3. Antioxidant Activities

#### 2.3.1. Total Antioxidant Capacity and Total Reducing Power

Results of TAC showed a wide range of values exhibited by plant extracts, expressed as the number of ascorbic acid equivalents, from 5.58 to 48.4 mg vit. C eq/g extract as shown in Table 3. *C. tamala* showed maximum TAC followed by *C. zeylanicum* and *M. piperita*. *C. sativum* displayed the lowest TAC. Furthermore, total reducing power results indicated a wide range of reducing power of extracts, ranging from 8.7 to 65.04 µg vit. C eq./mg Extract. The maximum TRP values were shown by *M. piperita* followed by *C. zeylanicum* and *C. tamala* (Table 3).

#### 2.3.2. DPPH Scavenging Assay

A percentage inhibition value was used to express the DPPH free radical scavenging activities of extracts. The inhibition values ranged from 60.9 to 84.7% for ethanol extract of selected plants at 1000 µg/mL concentration, as shown in Table 3. Free radical scavenging activity was observed in the *A. subulatum*, followed by *M. piperita* and *C. sativum.* Results revealed that plants showing the lowest IC_50_ values were *C. tamala, C. zeylanicum*, and *M. piperita.*

### 2.4. Phytochemical Investigation

#### 2.4.1. Total Phenolic and Flavonoid Content

The highest amount of TPC was quantified in *C. tamala* (176.5 ± 1.5 mg GAE/g Extract). Among ethanol extract of herbs, *M. piperita* showed the highest TPC (136.2 ± 6.4 mg GAE/g Extract), as presented in Table 3. Phenolic content declined in the following order *C. tamala* > *M. piperita* > *C. zeylanicum* > *A. subulatum* > *L. sativa Lactuca sativa* > *T. foenumgraecum* > *B. oleraceae* > *C. sativum*. Among spices, *T. foenumgraecum* represented the highest TFC while *M. piperita* showed the highest TFC among herbs (Table 3). The lowest TFC was detected in ethanolic extract of *A. subulatum* and *B. oleraceae.* The descending order of TFC among the extracts was *T. foenumgraecum > M. piperita > C. zeylanicum > L. sativum > C.tamala* *> C. sativum > B. oleraceae > A. subulatum*

#### 2.4.2. Reversed-Phase HPLC Analysis

**Figure 1:**
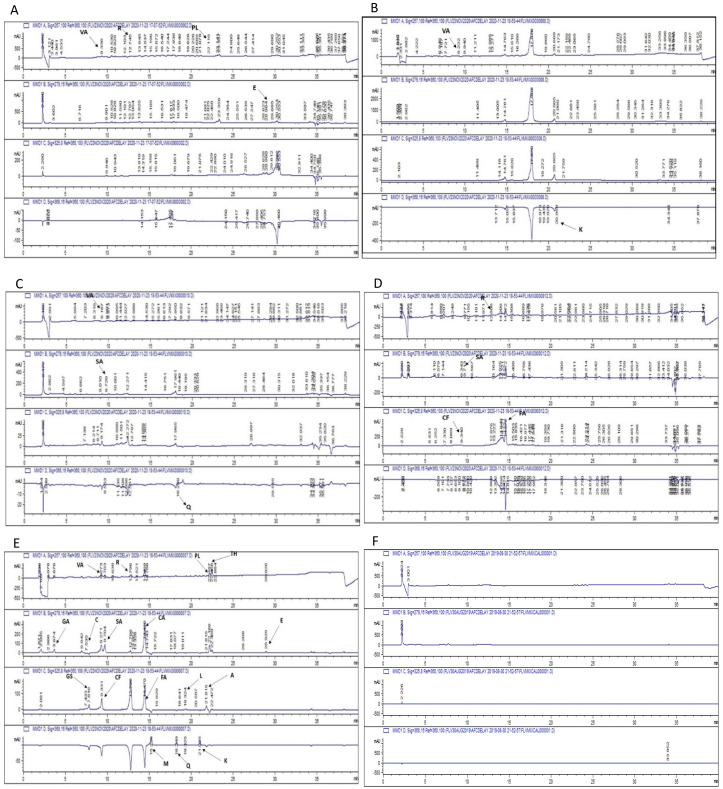
HPLC profile of (**A**) Ethanolic Extract of C.tamala; (**B**) Ethanolic extract of C.zeylanicum; (**C**) Ethanolic extract of A.subulatum;. (**D**) Ethanolic Extract of M.piperita; (**E**) Standard polyphenols; and (**F**) Blank. R; Rutin, VA; Vanillic acid, Q; Quercetin, FA; Ferulic acid, SA; Syringic acid, K; Kaempferol, PL; Plumbagion, C; Catechin, L; Luteolin, E; Emodin, CF; Caffeic acid, CA; Coumaric acid, and GS; Gentisic acid.

Quantitative polyphenol detection was carried out using HPLC (using a total of 18 standards), by comparing chromatograms of samples with that of standards (Table 4). A total of 13 polyphenols were detected in studied plant extracts, i.e., rutin, kaempferol, vanillic acid, quercetin, apigenin, ferulic acid, catechin, gentisic acid, syringic acid, plumbagin, caffeic acid, coumaric acid, and emodin. Rutin was found to be the most common polyphenol followed by vanillic acid, syringic acid, caffeic acid, quercetin and emodin. The highest amount of rutin (8.34 ± 0.26 µg/mg extract) and vanillic acid (2.35 ± 0.04 µg/mg extract) was found in *C. tamala*. While the maximum amount of ferulic acid (3.01 ± 0.26 µg/mg extract) and caffeic acid (0.43 ± 0.04 µg/mg extract) were quantified in *M. piperita*. Quercetin was found to be highest in *T. foenumgraecum* followed by *A. subulatum*. The representative chromatograms are shown in Figure 1.

### 2.5. Molecular Docking Analysis

**Figure 2:**
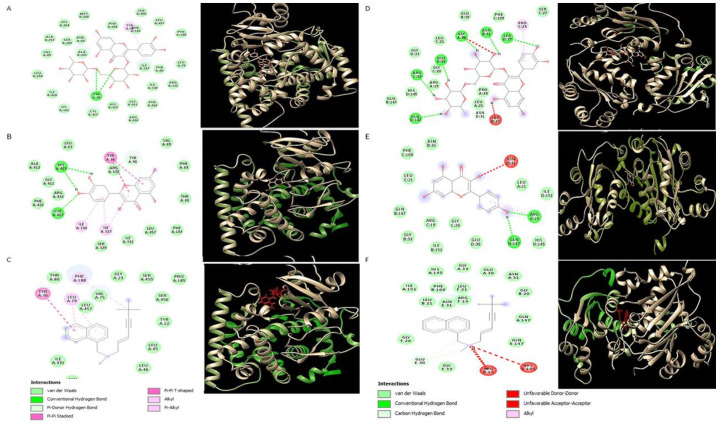
Graphical representation of polyphenols binding modes in fungal proteins. (**A**) Rutin with 14 alpha demethylase (CYP51). (**B**) Quercetin with CYP51. (**C**) Terbinafine with CYP51. (**D**) Rutin with nucleoside diphospho kinase (NDK). (**E**) Kaempferol with NDK. (**F**) Terbinafine with NDK.

To explore the underlying mechanism of antifungal activity, the docking interactions of common polyphenols were analyzed against two important fungal enzymes. Among all polyphenols, rutin showed the greatest affinity for 14-alpha demethylase (CYP51) and nucleoside diphosphokinase (NDK) with the lowest K_d_ (dissociation constant) values of −9.4 and −8.9, respectively (Table 5). The lower the K_d_ values, the better the binding affinity of a ligand would be with its target. Results showed that rutin interacted with fungal 14-alpha demethylase by forming hydrogen bonds with tyrosine 90 (Figure 2a), while, quercetin formed two hydrogen bonds with histidine 415 and cystine 417 (Figure 2b). Binding affinity was estimated to be −9.4 and −8 for rutin and quercetin, respectively (Table 5). Binding affinity for interaction with 14-alpha demethylase, were found to be increased in the following order: rutin > catechin > quercetin > kaempferol > vanillic acid > ferulic acid. Rutin and kaempferol showed the highest binding affinity for fungal nucleoside diphosphokinase with K_d_ −8.9 and −8.2, respectively. Rutin and kaempferol both interacted with nucleoside diphosphokinase by forming hydrogen bonds with arginine C:19 and glutamine D:147 (Figure 2d,e), while rutin also showed interaction with arginine C:19, glutamate: 30, glycine A: 20, aspergine: 21, and serine D: 27 (Figure 2d).

## 3. Discussion

Increasing incidence of drug-resistant fungi, the emergence of new fungal strains, and the toxicity profile of antifungal drugs have led to the use of medicinal plants as potential antifungal means. Further, understanding the mechanism by which plant antifungal agents, i.e., polyphenols, interact with crucial fungal proteins should be of prime importance in identifying the molecular targets [18]. Therefore, in the present research, crude ethanol extracts of selected plants, i.e., *C. zeylanicum*, *C. tamala, A. subulatum, T. foenumgraecum, M. piperita, C. sativum, L. sativa*, and *B. oleraceae var.italica* were prepared and screened for their antifungal activities, and polyphenols were identified in each plant. The polyphenols are strong antioxidants that have an important role in controlling microbial diseases. The respective polyphenols were further assessed for interaction with fungal proteins, via in silico analysis. Major findings of the current research are: (i) *C. zeylanicum, C. tamala*, and *A. subulatum* displayed significant antifungal activity among studied plants; (ii) rutin, kaempferol, and quercetin were identified as the most common antifungal polyphenols in selected plants; (iii) rutin displayed the greatest affinity with binding pocket of fungal 14-alpha demethylase (CYP51) and nucleoside diphosphokinase (NDK) with the lowest K_d_ values as compared to standard drug terbinafine; (iv) *C. zeylanicum* is predicted to possess antifungal activity due to kaempferol (inhibition of CYP51 and NDK; (v) *A. subulatum* and *T. foenumgraecum* antifungal effect are likely due to quercetin; and (vi): rutin was the major polyphenol found in *C. tamala* and all herbs, which might be responsible for antifungal activity by either competitive or allosteric inhibition of both studied fungal enzymes.

As per results of the current study, * C. zeylanicum* showed the highest percent yield in comparison to other plant extracts. Different plants exhibited different yields, perhaps due to differences in their phytochemical composition and their variable solubility [19]. Furthermore, ethanolic extracts *of C. zeylanicum, C. tamala,* and *A. subulatum* exhibited significant antifungal activities against different studied strains. A recent report has documented the antifungal efficacy of *C. zeylanicum* bark powder, its water suspensions, and its essential oils against *Fusarium oxysporum*. [20]. The *C. zeylanicum* methanol, *n*-hexane, and aqueous extracts are also reported to exhibit inhibitory effects against *Alternaria solani* [21]. To the best of our knowledge, there is no report presenting the antifungal effects of ethanol extract of *C. zeylanicum and A. subulatum* against *Fusarium solani*, which we observed in our study. We found that *C. zeylanicum* and *A. subulatum* ethanolic extracts only moderately inhibited the growth of both *Aspergillus* species. In a previous report, methanolic extract of *A. subulatum* exhibited a considerably higher zone of inhibition (i.e., 19 mm) against *Aspergillus niger* [22]. This might be due to different experimental conditions and different solvent system used. *T. foenumgraecum* is reported to inhibit the growth of fungal strains and exhibited inhibition effects against *Fusarium oxysporum* and *Fusarium. Oxysporum* [23]. In our study, the T. *foenumgraecum* has shown inhibition potential against *Fusarium solani* and *Aspergillus niger*. Extract of *M. piperita* inhibited the growth of two fungal strains, i.e., *Aspergillus niger* and *Fusarium solani*. *M. piperita* extract has been already shown to cause inhibitory effects against radial fungal growth and production of aflatoxin by *Aspergillus* species [24]. Moreover, the *B. oleraceae var.italica* in our study inhibited the growth of *Fusarium solani* and *Mucor spp*.

Next, our results revealed that plants having antifungal activities exhibited excellent antioxidant potential, evaluated by free radical scavenging assay, total antioxidant capacity, and total reducing power activity. The reducing power of *C. zeylanicum* leaf and bark extracts might be due to the di- and monohydroxyl substitutions in the aromatic ring, which possess potent hydrogen donating abilities as described by Shimada et al. [25]. Plants with high phenolic content are mostly targeted by pharmacists to treat infections [26]. It indicates that polyphenols are the actual contributor to the antioxidant and antifungal activity of the studied plants. In the current investigation, potent antifungal activities of *C. tamala*, *C. zeylanicum, A. subulatum*, and *M. piperita* were possibly observed due to their high phenolic content. The significant antioxidant potential of these plants might be the reason for their antifungal efficacy, as plant flavonoids are reported to inhibit biofilm formation by stimulating membrane disturbances, which reduces the fungal cell size and causes leakage of intracellular components [27]. Phenols are also oxidized in response to infection into their respective quinones which can further inactivate the fungal enzymes. Polyphenols have ortho para directing groups (which tend to donate electrons) which may contribute to their antioxidant as well as antifungal properties [28]. Hence, the findings of the current investigation support the previous views that, due to antioxidant compounds such as polyphenols, the plant extracts exhibit pronounced efficacy as an antimycotoxin [29,30].

To detect the common antifungal polyphenols from plants under study, we performed HPLC analysis. Kaempferol was detected in *C. zeylanicum*. In a previous study, HPLC chromatogram showed the presence of quercetin and kaempferol in cinnamon extract [31]. Polyphenol has been reported as justifiable resources for the control of fungal biofilms [32]. Kaempferol and quercetin seemed to be the contributor to the antifungal activity of *C. zeylanicum* and *A. subulatum* ethanol extracts, respectively. In addition, rutin might be involved in the antifungal action of all other test extracts. Rutin and quercetin are reported to enhance the antifungal activity of amphotericin B [33]. Quercetin has also been reported to exhibit individual or synergic antifungal properties with fluconazole (an inhibitor of fungal fatty acid synthase) [34]. Many studies presented the rutin as the potent agent for exhibiting the antifungal responses of the extracts [35]. Although phenolic compounds exhibit variable mechanisms for antimicrobial pharmacology, many of them act by promoting damage to the function of the cell membrane or cell wall [32,36]

To explore the underlying mechanisms inhibiting the fungal growth, in silico modeling was carried out which further confirmed the inhibition of fungal enzymes by the polyphenols detected in the extracts. We evaluated the inhibition potential of detected polyphenols against two fungal enzymes, i.e., CYP51 and NDK. The CYP51 belongs to the cytochrome P450 monooxygenase superfamily and mediates a crucial step of the synthesis of ergosterol, which is a fungal-specific sterol. The NDK catalyzes the reversible exchange of the *γ*-phosphate between nucleoside triphosphate (NTP) and nucleoside diphosphate (NDP) [37,38]. Among different reported antifungal mechanisms, rutin, and quercetin, found in our plant extracts, seem to inhibit fungal strains via inhibition of CYP51 enzyme, same as that of azole drugs. In a previous study, rutin was among the seven plant molecules showing excellent binding energy against CYP51 [39]. In the same study, quercetin formed five hydrogen bonds with HIS468, GLY307, THR311, LYS143, and TYR143, having binding energy −7.54 kcal/mol [39]. In our study, quercetin exhibited the binding energy of −8 kcal/mol to CYP51, which is in line with the previously mentioned finding. Many studies used terbinafine as a standard antifungal agent due to its efficacy against broad spectrum of pathogenic fungi [40]. As terbinafin also works by inhibition of ergosterol biosynthesis (via inhibition of squalene epoxidase) [41], in the present study, it was used as a control to compare the antifungal potential of plant polyphenols. Interestingly the K_d_ values of rutin (−9.4) were much lower than that of terbinafine (−8.9) which clearly indicates high inhibition potential of rutin for fungal CYP51 as compared to terbinafine. It may be as terbinafine and rutin both inhibit fungal ergostrol biosynthesis pathway but both work by inhibiting different enzymes of same pathway. In another report, fluconazole and ketozole showed binding affinity values of −7.6 and −10, respectively, with fungal alpha demethylase [42,43]. It indicates that rutin can inhibit CYP51 either alone or in combination with the above-mentioned drugs. Kaempferol revealed its antifungal potential by low K_d_ values against fungal NDK, which is reported for regulation of the spore development and pathogenicity [44]. Quercetin and rutin also showed a significant binding affinity with NDK, which attributes to their dual mechanism of action. The antifungal efficacy of these polyphenols also supports the structure-activity relationship analysis, recently revealed [28]. According to this study, phenolic compounds that do not support a conjugated π system have a low electrophilicity index, and compounds having a low electrophilicity index have good antifungal activities.

Other detected polyphenols such as vanillic acid, ferulic acid, catechin, and caffeic acid also have antifungal properties [45] but they might use an antifungal mechanism other than inhibiting these two fungal enzymes tested in the present study.

## 4. Materials and Methods

### 4.1. Plant Collection

Spices (*C. zeylanicum, C. tamala, A. subulatum*, and *T. foenumgraecum*) and herbs (*M. piperita, C. sativum, L. sativa,* and *B. oleraceae*) were collected during September 2020 from the local market of Bharakahu, Islamabad, Pakistan. The specimens were identified by Dr. Muhammad Zafar, Department of Plant Sciences, Quaid-i-Azam University, Islamabad.

### 4.2. Extract Preparation

The herbs were washed thoroughly under running tap water and dried under shade for three weeks. Dried spices and herbs were subjected to a fine powder and stored at room temperature in air-tight containers.Then, 200 g fine powder of each plant was macerated for five days, using analytical grade ethanol (1000 mL), in air-tight glass bottles. Maceration was performed to increase the contact between plant material and solvent (Ethanol) and to soften plant’s cell wall so that plant phytochemicals soluble in ethanol may be released. Sonication (after maceration) was added to further enhance the disruption of the plant cell wall and facilitate the release of phytochemicals. The extracts were filtered using Whatman #1 filter paper (Sigma, USA). Extracts were concentrated by vacuum evaporation in a rotary evaporator (Buchi, Switzerland) and dried in a vacuum hood at 40 °C, and stored at 15 °C till further use. The extracts were filtered using Whatman #1 filter paper. Extracts were concentrated by vacuum evaporation in a rotary evaporator (Buchi, Switzerland) and dried in a vacuum hood at 40 °C, and stored at 15 °C till further use.

The extracts, once dried, were weighed to find out % recovery by the following formula:

(1) $$\mathrm{Extract}\;\mathrm{recovery}\;\left(\%\frac{w}{w}\right)=\frac{A}{200}\times 100$$

where A = weight of dry extract.

### 4.3. Antifungal Activity

The antifungal activity of extracts was evaluated by the agar disc diffusion method, performed in triplicate [19]. The spores of test fungal strains Fusarium solani (FCBP-0291), Mucor species (FCBP-0300), Aspergillus niger (FCBP-0198), and Aspergillus flavus (FCBP-0064) were procured from the department of Pharmacy, Quaid-i-Azam University, Islamabad, Pakistan. These strains were collected in solution (0.02% *v*/*v* tween 20 in H_2_O) and the turbidity was adjusted according to McFarland 0.5 turbidity standard. Then, fungal strain (100 µL) was streaked on sabouraud dextrose agar. Discs of filter paper, impregnated with test extract (5 µL; 20 mg/mL), positive control (Terbinafine, 20 µg/mL), and negative control (DMSO) were employed on agar plates and incubated (24–48 h; 28 °C). Afterward, the average diameter (mm) of the growth inhibition zone around all the discs was measured and recorded.

### 4.4. Antioxidant Activities

#### 4.4.1. Total Reducing Power (TRP)

The reducing power assay was performed as described by Ahmed et al., with some modifications [19]. Briefly, test extract (50 μL; 20 mg/mL in DMSO) was mixed with 475 μL of phosphate buffer (0.2 mol/L, pH 6.6) and potassium ferricyanide (1% *w*/*v* in H_2_O). The mixture was incubated (20 min; 50 °C) and then 500 μL of trichloroacetic acid was added. Afterward, centrifuged at room temperature (10 min). The upper layer (500 μL) was mixed with eqal volumn of distilled water and FeCl_3_ (100 μL; 0.1% *w*/*v* in H_2_O). Then, 200 μL was takenfrom this was taken and the absorbance of the was measured at 645nm using microplate reader (Biotech, Elx-800, St. Louis, MO, USA). The assay was performed in triplicate. The chemicals and standards used in the assays were purchased from Sigma-Aldrich (St. Louis, MO, USA)**.**

#### 4.4.2. Total Antioxidant Capacity (TAC)

Total antioxidant capacity was determined by the phosphomolybdenum method [46]. An aliquot of 10 µL extract (20 mg/mL in DMSO) was added to 190 µL of reagent solution (0.6 M sulphuric acid, 28 mM sodium phosphate, and 4 mM ammonium molybdate solution in H_2_O) in 96 well plate. After incubation for 90 min at 95 °C in the water bath, the reaction mixture was cooled at room temperature and absorbance was taken at 630 nm using microplate reader (Biotech, Elx-800, USA). Both TRP and TAC were expressed as the number of mg of vitimin C (vit. C) equivalents per gram of extract (vit. C eq/g). The chemicals and standards used in the assay were purchased from Sigma-Aldrich.

#### 4.4.3. DPPH (2,2-Diphenyl1–1-picryl-hydrazyl radical) Free Radical Scavenging

Discoloration of purple-colored DPPH was used to measure the free radical scavenging activities of extracts [19,46]. Here, 190 µL of DPPH solution (in methanol) was added in each well (96 well plate) followed by the addition of 10 µL of four different dilutions (125, 250, 500, and 1000 µg/mL) of each extract. Samples were incubated in the dark at 37 °C for 15 min and absorbance was measured at 517 nm using microplate reader (Biotech, Elx-800, St. Louis, MO, USA). The assay was performed in triplicate and ascorbic acid was used as a positive control in each experiment. Percentage free radical scavenging was derived from the given formula:

(2) % Scavenging=Absorbance of Negative control - Absorbance of extract/Absorbance of negative control × 100

The inhibitory concentration at which the test sample showed 50% scavenging (IC_50_) was determined using Graph Pad Prism 5 software.

### 4.5. Phytochemical Analysis

#### 4.5.1. Total Phenolic Content (TPC)

TPC was measured by the Folin–Ciocalteu method as reported previously [47]. Briefly, 10 µL (20 mg/mL in DMSO) of each extract was mixed with Folin–Ciocalteu reagent (10 fold diluted with water). After 5 min of incubation at room temperature, 98 µL of 6% sodium bicarbonate was added. The resulting mixture was kept at 25 °C for 90 min and absorbance was measured at 630 nm using microtitre plate reader. The standard curve equation for gallic acid was obtained (y = 0.0732x − 0.0205), with R^2^ value of 0.999. The assay was performed in triplicate and TPC was determined as mg of gallic acid equivalent (GAE) per gram of extract (mg GAE/g Extract). The standard gallic acid and other chemicals used in this assay were purchased from Sigma-Aldrich.

#### 4.5.2. Total Flavonoid Content (TFC)

The TFC of the extracts was determined by aluminium chloride colorimetric assay, with modifications [19]. Briefly, from each extract stock (10 μL; 20 mg/mL in DMSO), aluminium chloride (10 μL; 10% *w*/*v* in H_2_O), 1.0 M potassium acetate (10 μL), and distilled water (170 μL) were mixed and incubated at room temperature (30 min). The absorbance of the mixture was calculated at 405 nm.

The resultant TFC was expressed in microgram equivalents of quercetin per milligram Extract (µg QE/mg Extract). The standard curve equations for quercetin (Sigma-Aldrich) (y = 0.01x − 0.004) and the R^2^ value of 0.996 were obtained.

#### 4.5.3. High-Performance Liquid Chromatography (HPLC) Analysis

Polyphenols were detected and quantified using HPLC analysis of medicinal plants. HPLC system, Agilent Chem station Rev. B.02–01-SR1 (260), equipped with a Zorbex-C8 analytical column (4.6 × 250 nm, 5 μm particle size) in combination with a diode array detector (DAD; Agilent technologies, Waldbronn, Germany) was used, following previously reported method [18]. All HPLC standards were purchased from Sigma Aldrich. Wavelengths used to detect the standards were 257 nm for rutin, vanillic acid, and plumbagin, 279 nm for catechin, syringic acid, coumaric acid, and emodin, 325 nm for caffeic acid, apigenin, luteolin, gentisic acid, and ferulic acid, while quercetin and kaempferol were analyzed at 368 nm. Different polyphenols were identified and quantified by comparing the UV absorption spectra and the retention time of samples was compared with those of standards and the results were expressed as μg/mg extract.

### 4.6. Molecular Docking

Molecular docking was performed to predict the possible antifungal mechanisms of polyphenols, detected via HPLC analysis. Protein databank (PDB) was used to obtain 3D structures of fungal enzymes 14-alpha-demethylase and nucleoside diphosphokinase with PDB IDs 5FRB and 6K3H, respectively. A 3D structure CYP51B protein was modeled using Swiss Prot. Pubchem was used to obtain structures of ligands (i.e.: rutin, quercetin, apigenin, and kaempferol with PubChem IDs 5280805, 5280343, 5280443, and 5280863, respectively). Ligands and heteroatoms were removed and proteins were optimized and minimized using UCSF Chimera software to obtain structurally correct protein. Docking was performed using the Pyrx-virtual screening tool, binding affinities were saved as CSV files and Discovery studio was used to visualize the final protein–ligand interactions [48].

### 4.7. Statistical Analysis

Statistical analysis was carried out using SPSS statistics and MS Excel. Replication of each experiment was conducted thrice.

## 5. Conclusions

This study revealed new scientific insights about the antifungal activity of natural polyphenols and their mechanism of action. *C. zeylanicum, C. tamala,* and *A. subulatum* represent good sources of such antifungal polyphenols. Bio-formulation involving extracts from more than one plant may be necessary for effective antifungal application. Our study suggested that high-phenolic content of plant extracts is responsible for their antioxidant and fungal-inhibitory activity. These tested plants may be used for developing new, safer, and effective fungicides. Moreover, detected polyphenols can be used either alone or synergistically with the fungal antibiotics to reduce their toxic effects and to increase the antifungal efficacy.

## Figures and Tables

**Table 1:** Percent extract recovery of plants using methanol for extraction. Extraction was executed in triplicate and the data are presented as mean ± standard deviation (SD).

Plant Name	Percent Yield (%)
*C. zeylanicum*	26.51 ± 2.22
*C. tamala*	18.79 ± 1.22
*A. subulatum*	9.45 ± 1.11
*T. foenumgraecum*	5.075 ± 0.52
*M. piperita*	10.275 ± 1.11
*C. sativum*	5.995 ± 0.91
*L. sativa*	6.37 ± 0.81
*B. oleraceae*	7.025 ± 1.02

**Table 2:** Antifungal activity against tested strains at 100 µg/disc concentration. Experiments were executed in triplicate and the data are presented as mean ± standard deviation (SD).

-	Zone of Inhibition (mm)			
-	*Fusarium solani*	*Aspergillus niger*	*Aspergillus flavus*	*Mucor* Spp.
*C. zeylanicum*	13.0 ± 0.5	10.0 ± 0.1	8.1 ± 0.1	10.0 ± 0.1
*C. tamala*	11.3 ± 1.0	-	-	7.1 ± 0.1
*A. subulatum*	13.6 ± 0.1	7.1 ± 0.1	5.0 ± 0.0	-
*T. foenumgraecum*	9.3 ± 0.0	7.1 ± 0.2	-	-
*M. piperita*	10.0 ± 0.1	6.0 ± 0.1	-	-
*C. sativum*	7.6 ± 0.3	-	-	-
*L. sativa*	9.3 ± 0.4	-	-	-
*B. oleraceae*	8.3 ± 0.5	-	-	9.2 ± 0.0
Terbinafine	20 ± 0.9	22 ± 1.03	23 ± 1.0	22 ± 1.0

*Cinnamomum zeylanicum (C. zeylanicum), Cinnamomum tamala (C. zamala), Amomum subulatum (A. subulatum), Trigonella foenum graecum (T. foenumgraecum), Mentha piperita (M. piperita), Coriandrum sativum (C.sativum),* and *Brasicca oleraceae var. italica (B. oleraceae).*

**Table 3:** Antioxidant potential and total phenolic and flavonoid content of ethanol extracts of selected plants. Experiments were executed in triplicate and the data are presented as mean ± standard deviation (SD).

S.No	Plant Name	Total Reducing Power (Vit C Equiv mg/g Extract)	Total Antioxidant Capacity (Vit C Equiv mg/g Extract)	DPPH Free Radical Scavanging (%)		Total Phenolic Content (mg GAE/g Extract)	Total Flavonoid Content (mg QE/g Extract)
-	-	-	-	% Scavanging at 1000 ppm (%)	IC50 (mg/mL)	-	-
1	*C. zeylanicum*	63.08 ± 0.22	36.75 ± 0.63	78.82 ± 2.52	25.4	82.42 ± 5.62	23.66 ± 0.13
2	*C. tamala*	57.72 ± 0.41	48.47 ± 0.91	82.93 ± 2.11	8.681	176.51 ± 1.52	13.69 ± 0.32
3	*A. subulatum*	8.48 ± 2.42	29.97 ± 0.71	84.73 ± 3.51	283.4	24.42 ± 0.07	2.45 ± 0.42
4	*T. foenumgraecum*	9.28 ± 2.31	21.20 ± 0.82	75 ± 3.32	485.9	25.51 ± 0.34	28.79 ± 0.38
5	*M. piperita*	65.04 ± 1.11	32.73 ± 0.82	83.42 ± 3.52	124.9	136.22 ± 6.41	26.78 ± 1.11
6	*C. sativum*	15.72 ± 0.82	5.58 ± 2.12	83.52 ± 4.52	250.9	25.17 ± 0.82	11.56 ± 0.57
7	*L. sativa*	23.22 ± 0.21	14.56 ± 1.06	80.77 ± 5.52	221.6	28.63 ± 3.22	15.28 ± 1.29
8	*B. oleraceae*	8.72 ± 2.85	21.89 ± 1.11	60.99 ± 2.53	759.4	17.17 ± 3.22	2.47 ± 1.11

**Table 4:** Identification and quantification of polyphenols (µg/mg Extract) in ethanol extracts of selected plants by HPLC. Experiments were executed in triplicate and the data are presented as mean ± standard deviation (SD).

S.N	Plant	Rutin (R)	Vanillic Acid (VA)	Quercetin (Q)	Ferulic Acid (FA)	Syringic Acid (SA)	Kaempferol (K)	Plumbagin (PL)	Apigenin (A)	Catechin (C)	Luteolin (L)	Emodin (E)	Caffeic Acid (CF)	Coumaric Acid (CA)	Gentisic Acid (GS)
1	*C. zeylanicum*	-	0.21 ± 0.02	-	-	-	0.63 ± 0.02	-	-	-	-	-	-	-	-
2	*C. tamala*	8.34 ± 0.26	2.35 ± 0.04	-	-	-	-	0.11 ± 0.04	-	-	-	0.54 ± 0.03	-	-	-
3	*A. subulatum*	-	0.21 ± 0.01	0.85 ± 0.03	-	0.2 ± 0.04	-	-	-	-	-	-	-	-	-
4	*T. foenumgraecum*	6.32 ± 0.03	-	1.35 ± 0.04	-	0.24 ± 0.06	-	-	0.53 ± 0.03	-	-	0.05 ± 0.002	-	--	-
5	*M. piperita*	4.3 ± 0.05	-	-	3.01 ± 0.26	0.22 ± 0.07	-	-	-	-	-	-	0.43 ± 0.04	-	-
6	*C. sativum*	3.44 ± 0.06	-	-	-	-	-	-	-	-	-	-	0.27 ± 0.02	-	1.01 ± 0.22
7	*L. sativa*	3.07 ± 0.11	-	-	-	-	-	-	-	0.55 ± 0.03	-	-	0.37 ± 0.04	-	-
8	*B. oleraceae*	0.84 ± 0.03	-	-	-	-	-	-	-	-	-	-	-	-	-

**Table 5:** Binding affinities of polyphenols interaction with fungal proteins in terms of K_d_ values using molecular docking analysis.

Polyphenol	14-alphaDemethylase (CYP51)	Nucleoside Diphosphokinase (NDK)
Rutin	−9.4	−8.9
Quercetin	−8	−7.8
Kaempferol	−7.9	−8.2
Vanillic acid	−5.7	−5.6
Ferulic acid	−6.1	−5.9
Catechin	−8.1	−7.7

## Data Availability

The datasets used and/or analyzed during the current study are available from the corresponding author on reasonable request.

## Sample Availability

Samples of plant material are available from the authors.
